# Efficient Cross-Screening and Characterization of Monoclonal Antibodies against Marek’s Disease Specific Meq Oncoprotein Using CRISPR/Cas9-Gene-Edited Viruses

**DOI:** 10.3390/v15040817

**Published:** 2023-03-23

**Authors:** Man Teng, Jin-Ling Liu, Qin Luo, Lu-Ping Zheng, Yongxiu Yao, Venugopal Nair, Gai-Ping Zhang, Jun Luo

**Affiliations:** 1 Key Laboratory of Animal Immunology, Ministry of Agriculture and Rural Affairs of China and Henan Provincial Key Laboratory of Animal Immunology, Henan Academy of Agricultural Sciences, Zhengzhou 450002, China; 2 UK-China Centre of Excellence for Research on Avian Diseases, Henan Academy of Agricultural Sciences, Zhengzhou 450002, China; 3 College of Animal Science and Technology, Henan University of Science and Technology, Luoyang 471003, China; 4 College of Veterinary Medicine, Henan University of Animal Husbandry and Economy, Zhengzhou 450046, China; 5 The Pirbright Institute & UK-China Centre of Excellence for Research on Avian Diseases, Pirbright, Ash Road, Guildford GU24 0NF, UK; 6 International Joint Research Center of National Animal Immunology and College of Veterinary Medicine, Henan Agricultural University, Zhengzhou 450002, China; 7 Jiangsu Co-Innovation Center for Prevention and Control of Important Animal Infectious Disease and Zoonoses, Yangzhou University, Yangzhou 225009, China

**Keywords:** herpesvirus, MDV, Meq, CRISPR/Cas9, gene editing, monoclonal antibody

## Abstract

Marek’s disease (MD) caused by pathogenic Marek’s disease virus type 1 (MDV−1) is one of the most important neoplastic diseases of poultry. MDV−1-encoded unique Meq protein is the major oncoprotein and the availability of Meq-specific monoclonal antibodies (mAbs) is crucial for revealing MDV pathogenesis/oncogenesis. Using synthesized polypeptides from conserved hydrophilic regions of the Meq protein as immunogens, together with hybridoma technology and primary screening by cross immunofluorescence assay (IFA) on Meq-deleted MDV−1 viruses generated by CRISPR/Cas9-gene editing, a total of five positive hybridomas were generated. Four of these hybridomas, namely 2A9, 5A7, 7F9 and 8G11, were further confirmed to secrete specific antibodies against Meq as confirmed by the IFA staining of 293T cells overexpressing Meq. Confocal microscopic analysis of cells stained with these antibodies confirmed the nuclear localization of Meq in MDV-infected CEF cells and MDV-transformed MSB-1 cells. Furthermore, two mAb hybridoma clones, 2A9-B12 and 8G11-B2 derived from 2A9 and 8G11, respectively, displayed high specificity for Meq proteins of MDV−1 strains with diverse virulence. Our data presented here, using synthesized polypeptide immunization combined with cross IFA staining on CRISPR/Cas9 gene-edited viruses, has provided a new efficient approach for future generation of specific mAbs against viral proteins.

## 1. Introduction

Marek’s disease (MD), caused by the pathogenic Marek’s disease virus (MDV), can produce rapid-onset T-cell lymphomas and immunosuppression in its natural chicken hosts and lead serious economic losses to the poultry industry worldwide [1]. As important members of the subfamily *Alphaherpesvirinae* in the family *Herpesviridae*, the MD-associated avian herpesviruses include three types: MDV type 1 (MDV−1) or *Gallid alphaherpesvirus 2* (GaAHV-2), MDV type 2 (MDV−2) or *Gallid alphaherpesvirus 3* (GaAHV-3) and Herpesvirus of turkeys (HVT) or *Meleagrid alphaherpesvirus 1* (MeAHV-1) [2]. Only the virulent MDV−1 strains are pathogenic and/or oncogenic to the hosts. Based on the mortality in chicken flocks, lesion frequency, and immune-protection, MDV−1 field strains have been further grouped into distinct pathotypes, such as mild MDV (mMDV), virulent MDV (vMDV), very virulent MDV (vvMDV), and very virulent plus MDV (vv+MDV) [3]. The clinical symptoms and development of MD tumors can be successfully prevented by vaccination with nonpathogenic or attenuated MDV strains, which have provided excellent models for the study of vaccination approaches in virally induced cancer biology [4]. In recent years, due to the continuous emergence of MDV−1 epidemic strains with increasing virulence and the widespread genovariations caused by continuous immune pressure of universal MD vaccination in chicken flocks, MD outbreak to the global poultry industry has never been eliminated [5,6,7,8]. 

MDV−1 encodes a number of viral protein-coding genes and non-coding RNA genes, some of which contribute a lot to MDV pathogenesis and tumorigenesis [9,10,11]. As the major oncoprotein known to date, Meq is the most highly variable protein among nearly 100 viral proteins encoded by MDV−1. The evolutionary rate of Meq is much faster than those of most double-strand DNA viruses, and is equivalent to that of RNA viruses [12]. Amino acid variations of Meq do exist among different MDV−1 epidemic strains, even among different strains within the same pathotypes that show genetic diversity and varying virulence. Therefore, Meq has always been regarded as the preferred target for studying evolution of virulence among MDV−1 epidemic strains [5]. Meq contains nuclear and nucleolar localization signals, a basic-region leucine zipper (bZIP) domain and a proline rich transactivation/repression domain, and is consistently expressed in all tumor and latently infected cells [13,14]. As the bZIP domain of Meq shares homology with other oncoproteins such as the Fos/Jun family and deletion of Meq gene resulted in the loss of T-cell transformation in chickens, Meq has been characterized as the major oncoprotein of MDV [14,15]. 

For a long time, the molecular mechanism of MD tumorigenesis induced by Meq has been an attractive research topic, hence monoclonal antibodies (mAbs) against Meq are becoming crucial. In previous studies [16,17,18,19], using MDV-infected CEF culture as immunogens, only a limited number of MDV-specific mAbs had been developed and prepared in the 1980s–1990s, but identities and potential usages of these mAbs had not been well defined. In 1997, the Meq mAbs had been first prepared using recombinant rFPV/Meq protein as immunogens due to the low expression of Meq in MDV infected CEF cells [20]. Two more Meq-specific mAbs have been successfully generated subsequently and the recombinant proteins were used as immunogens in both cases. However, the usage of these mAbs had only been restricted to immunofluorescence assay (IFA) or immunohistochemistry (IHC) analysis and their cross-reactivity to different MDV strains remains unclear [20,21,22,23]. Therefore, in the present study, we have analyzed the amino acid conservation and hydrophobicity of Meq proteins among MDV−1 strains such as 648A, Md5, GX0101 and GA that differ in virulence. We then synthesized polypeptides from conserved and soluble regions to be used as antigens after coupling with Keyhole limpet hemocyanin (KLH) for immunization of Balb/C mice and production of hybridomas. To characterize the specific mAbs produced by synthetic Meq polypeptide, we cross-screened hybridomas secreting Meq-specific antibodies by IFA using vvMDV strains Md5, GX0101 and their Meq deletion mutants previously generated using CRISPR/Cas9-based gene editing system [24]. As expected, our data exemplified a new strategy for the efficient screening and characterization of specific mAbs against MDV−1 Meq proteins, providing a new approach for the future generation of other virus-specific mAbs.

## 2. Materials and Methods

### 2.1. Ethics Statement

The animal experiment was approved by the Laboratory Animal Management Committee of Key Laboratory of Animal Immunology, Ministry of Agriculture and Rural Affairs, China, following the protocols of the Laboratory Animal-Guideline for Ethical Review of Animal Welfare permitted by the State Administration for Market Regulation and Standardization Administration of China (permit no. GB/T 35892-2018). 

### 2.2. Viruses and Cells

The mutants Md5∆meq and GX0101∆meq lacking the Meq genes of vvMDV strains Md5 and GX0101 were previously constructed in our laboratory using the CRISPR/Cas9 system [24]. The other virulent or vaccine strains of MDV including Md5, GX0101, GA, CVI988, SB-1 and HVT were used for detection of the reactivity spectrum of mAbs. Primary chicken embryo fibroblasts (CEFs) were prepared from 10-day-old SPF chicken embryos (Boehringer Ingelheim Vital Biotechnology Co., Ltd., Beijing, China). The CEF monolayers were maintained in M199 medium (Gibco, New York, NY, USA) supplemented with 5% fetal bovine serum (FBS) (Sigma, Losis, MO, USA), 10% tryptose phosphate broth (TPB) (Bacto, Sparks, NV, USA), 100 units/mL of penicillin and 100 µg/mL streptomycin (Ncmbio, Suzhou, China). The 293T cells were cultured in Dulbecco’s Modified Eagle’s Medium (DMEM) (Gibco, New York, NY, USA) containing 10% FBS and penicillin-streptomycin. All cells and viruses were incubated at 38.5 °C in a 5% CO_2_ incubator. 

### 2.3. Analysis of Amino Acid Sequence and Synthesis of Meq Polypeptide

The sequence alignment, conservation and hydrophilic regions of Meq amino acid sequences from MDV−1 strains 648A, Md5, GX0101 and GA were analyzed using the biological software DNAMAN (Lynnon Biosoft, San Ramon, CA, USA), MegAlign (DNAStar, Madison, WI, USA) and the online tool Expasy (Expert Protein Analysis System, http://www.expasy.ch) posted on 15 March 2023—Release 126 of Rhea by the Swiss Institute of Bioinformatics (Lausanne, Switzerland). The polypeptides of hydrophilic regions, as listed in Table 1, were synthesized and coupled with KLH (GL Biochem Shanghai Ltd., Shanghai, China). 

### 2.4. Production of Antibodies against Meq and Primary IFA Analysis

Six-week-old female Balb/C mice were subcutaneously immunized with the equally mixed polypeptide-KLH antigens (50 μg/mouse in Freund’s adjuvant) at 3-week intervals for 3 times. The final immunization was performed with the same dose of antigen (50 μg in 100 μL PBS) at 3 days before cell fusion. Conventional cell fusion technology was employed to generate hybridomas. After 7–10 days post fusion, supernatant of hybridomas was collected for IFA staining. The CEF monolayers grown in 96-well plates were infected with GX0101 viruses for 48–72 h before staining. After being fixed with pre-chilled methanol/acetone (*v*/*v* = 1:1) and blocked with 5% skimmed milk, the infected cells were stained with the culture supernatant of hybridomas, followed by incubation with the secondary antibody DyLight 488 labeled Goat Anti-Mouse IgG (1:1000) (Abbkine, CA, USA). Images were taken using inverted fluorescence microscopy (Zeiss, Jena, Germany). 

### 2.5. Screening and Characterization of Meq-Specific Antibodies

The CEF monolayers in 24-well plates were infected with Md5, GX0101 or their Meq-deleted mutant viruses to produce MDV plaques as previously described [24], and then fixed and incubated with positive candidates of Meq mAb to perform IFA staining as described above. The ones showing specific positive reaction to Md5 and GX0101 plaques but not to Md5∆meq or GX0101∆meq were confirmed as positive hybridomas specific to MDV−1 Meq proteins by observation under fluorescence microscopy (Zeiss, Jena, Germany).

### 2.6. Transient Expression of Meq and Examination of Antibody Specificity

The full-length Meq gene sequence of vvMDV strain Md5 was optimized, synthesized and cloned into the eukaryotic expression plasmid pEGFP-N1 (SunYa, Hangzhou, China) to generate the pEGFP-N1-meq plasmid. The recombinant or empty plasmids were separately transfected into 293T cells in 24-well plate using Lipofectamine^TM^ 2000 (Thermo Fisher Scientific, Basingstoke, UK) according to the manufacturer’s instruction. Two days later, the cells were fixed with pre-chilled methanol/acetone (*v*/*v* = 1:1) and blocked with 5% skimmed milk, and then incubated with the Meq mAb candidates and the secondary antibody DyLight 594 labeled Goat Anti-Mouse IgG (Abbkine, CA, USA) sequentially. The anti-Meq mAb FD7 (produced in The Pirbright Institute, Surrey, UK) serves as a positive control for Meq. Images were taken using an inverted fluorescence microscopy (Zeiss, Jena, Germany).

### 2.7. Confocal Microscopic Analysis

The expression of MDV−1 Meq and gB proteins in virus-infected CEFs and MDV-transformed MSB-1 cells was detected by IFA staining and visualized using confocal laser scanning microscope. CEF cells infected with GX0101 for 2 days were trypsinized and transferred to special confocal cell culture plate at low density, incubated at 38.5 °C in a 5% CO_2_ incubator for 24 h. Each of 1 × 10^6^ of MSB-1 cells was adhered to the special confocal cell culture plate with Cell and Tissue Adhesive (Corning^®^ Cell-Tak^TM^, Bedford, MA, USA). Following the IFA staining protocol as described above, the cells were stained with the supernatant of positive hybridomas and DyLight 594 labelled Goat Anti-Rabbit IgG (Abbkine, CA, USA) sequentially, followed by incubation with the anti-gB mAb HB3 (produced in The Pirbright Institute, UK) and DyLight 488 labelled Goat Anti-Mouse IgG (Abbkine, CA, USA) in the same way. Cell nuclei were stained with DAPI (4,6-diamidino-2-phenylindole). Images were taken using confocal laser scanning microscope (Zeiss, Jena, Germany).

### 2.8. Subtype Characterization of Meq-Specific mAbs

The hybridomas secreting the Meq-specific mAbs with the stronger reactivity and higher titers were further cloned and purified by limiting dilution. The ascitic fluids from the positive monoclonal hybridomas were produced in multiparous Balb/C mice and the titers were determined by IFA staining, as described above. The subtype of each mAb was determined using the Mouse Monoclonal Antibody Isotyping Kit (Proteintech).

### 2.9. Reaction Spectrum of Meq-Specific mAbs to MDVs

To investigate the reaction specificities of newly developed Meq mAbs to different virulence of MDVs and HVT, the CEF monolayers were infected with GX0101, Md5, GA, CVI988, SB-1 or HVT viruses separately to produce virus plaques, and the cells were fixed and incubated with ascitic fluids of the newly identified Meq mAbs, diluted in 1:5000 or 1:2000 for 2A9-B11 and 8G11-B2, respectively, to perform IFA staining. Simultaneously, the virus-infected CEF cells were collected and boiled with 1 × SDS-PAGE Sample Loading Buffer (Beyotime) for 10 min. The samples were separated on Bolt^TM^ Bis-Tris Plus 4–12% precast gel (Invitrogen), and the resolved proteins were then transferred onto PVDF membranes by iBlot^®^ 2 PVDF Regular Stacks (Invitrogen). The expression levels of Meq in each virus-infected CEFs were determined by incubation of newly identified Meq mAb and HRP-labeled Goat Anti-Mouse IgG (Pharmacia) sequentially and were finally visualized using NcmECL Ultra (NCM). In all cases, the chicken β-actin (Sangon Biotech, Shanghai, China) serves as the loading control.

## 3. Results

### 3.1. Amino Acid Sequence Analysis of the MDV−1 Meq Proteins

**Figure 1:**
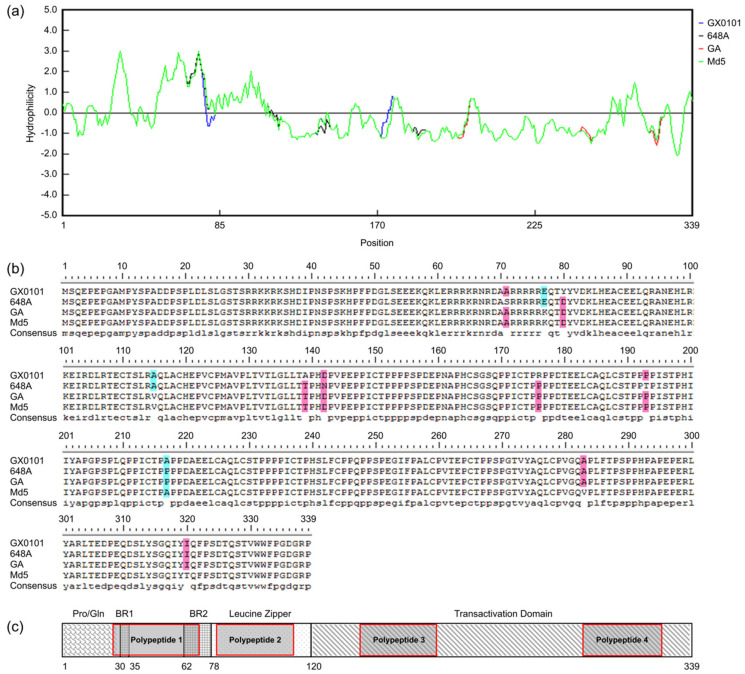
Amino acid sequence alignment, hydrophilicity and molecular structure analysis of the Meq proteins. (**a**) Comparison of amino acid hydrophilicities of different virulence MDV−1 Meq proteins. The upper half of the baseline represents the hydrophilic regions. The green curve represents the Md5 strain used as reference. The blue, black and red curves represent the amino acid difference region of GX0101, 648A and GA strains relative to Md5 strain, respectively. (**b**) Amino acids point mutation analysis of the Meq proteins of four MDV−1 strains with different virulence. Blue and pink indicate the mutant amino acids of different strains. (**c**) The genomic location and domain diagram of MDV−1 Meq protein. The relative positions of synthetic polypeptides are shown by shaded grey boxes with red brackets.

In order to design soluble polypeptides for the conserved regions of MDV−1 Meq proteins for use as immunogens, we have downloaded the Meq gene sequences of representative strains including vv+MDV (648A), vvMDV (Md5 and GX0101) and vMDV (GA) from the NCBI database, and used three different bio-softwares to compare and analyze the hydrophilicity and homology of Meq amino acid sequences of these MDV−1 strains. We found that the hydrophilicity of the Meq proteins of the four strains were exactly the same, and the hydrophilic regions were mainly clustered in amino acids 26–113, 141–183, and 285–315 (Figure 1A). There were only 11 amino acid differences in the Meq proteins of the four strains (Figure 1B). Therefore, combined with the genomic location and domain structure of Meq (Figure 1C), 4 polypeptides avoiding amino acid mutation sites in hydrophilic regions of Meq, as listed in Table 1, were selected and synthesized.

### 3.2. Generation of Candidate Antibodies against the Meq Proteins

To generate mAbs specific for Meq of MDV−1, the synthesized polypeptides from hydrophilic regions of Meq were coupled to KLH and used for immunizing female Balb/C mice. Three weeks post the third immunization, the tail vein blood of immunized mouse was collected and the presence of Meq antibody was determined by IFA staining with the viral plaques produced in GX0101-infected CEF cells. The result has shown that only some of the immunized mice displayed specific and high titers of serum polyclonal antibodies against MDV plaques. These mice were selected for cell fusion with conventional hybridoma technology and supernatant of hybridomas was screened by IFA staining. Finally, the supernatants from five positive hybridomas, namely 2A9, 4F3, 5A7, 7F9 and 8G11, were primarily detected to be positive for MDV plaques.

### 3.3. Screening and Identification of Specific Antibodies for Meq

**Figure 2:**
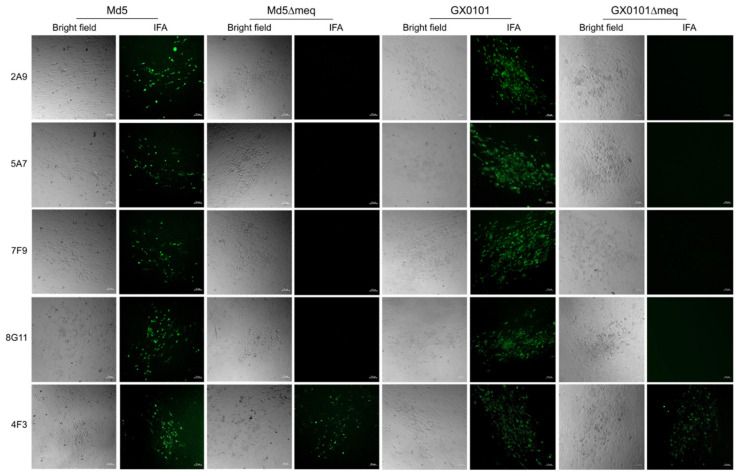
Cross screening and identification of Meq-specific antibodies by immunofluorescence assay. Plaques produced in CEFs by the infection with parental or Meq-deleted viruses were stained with candidate Meq antibodies and visualized by immunofluorescence microscope. Bright field, images of MDV plaques under regular light; IFA, immunofluorescence assay. (Scale bar = 50 µm).

**Figure 3:**
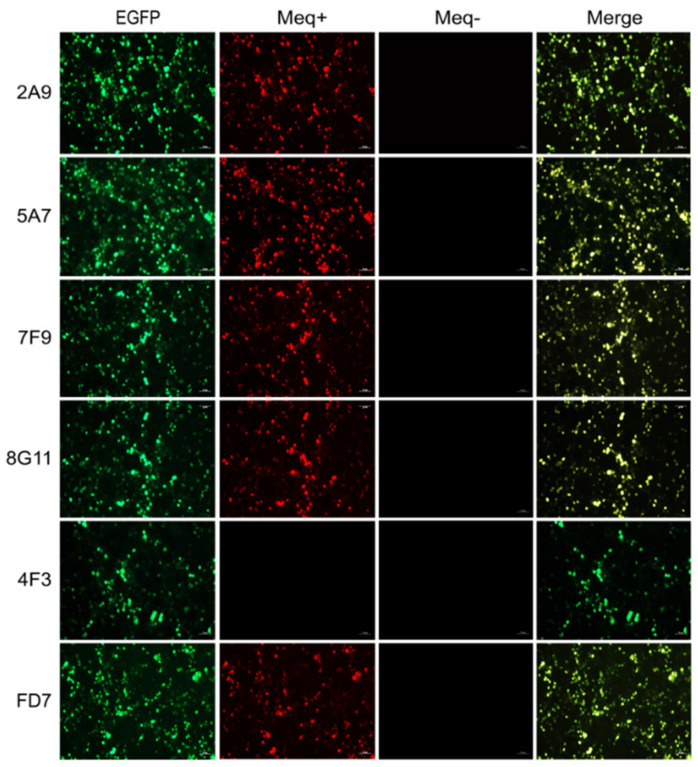
Staining of the Meq proteins overexpressed in 293T cells by immunofluorescence assay. The 293T cells transfected with the recombinant or empty plasmids, pEGFP-N1-Meq (Meq+) and pEGFP-N1 (Meq-), were fixed at 48 h post transfection, and then incubated with the candidate Meq antibodies and DyLight 594 labeled Goat Anti-Mouse IgG sequentially. The anti-Meq mAb FD7 served as a positive control. EGFP, enhanced green fluorescent protein with auto-fluorescence in green; Meq, MDV−1 specific oncoprotein stained in red; Merge, merged image in yellow. (Scale bar = 50 µm).

For the screening and identification of specific antibodies for MDV−1 Meq protein, the reactivity of 5 positive hybridomas mentioned above was checked by cross IFA staining of the virus plaques produced in Md5, Md5∆meq, GX0101 or GX0101∆meq-infected CEF monolayers. As demonstrated in Figure 2, the supernatant of hybridomas 2A9, 5A7, 7F9 and 8G11, was observed to specifically react to the viral plaques formed in Md5 or GX0101-infected CEFs but not react with those produced by Md5∆meq or GX0101∆meq viruses. However, supernatant of hybridoma 4F3 reacted with the viral plaques formed by both of the parental and meq-deleted viruses. For further confirmation of specificity of these antibodies, the 293T cells were transfected with pEGFP-N1-Meq plasmid to express Meq and the reactivity of supernatant from these five hybridomas to recombinant proteins were detected by IFA. As demonstrated in Figure 3, the IFA staining showed that the overexpressed Meq were specifically stained in red by supernatant of the hybridomas 2A9, 5A7, 7F9 and 8G11. The expressed Meq (in red) was co-localized with the spontaneous EGFP green fluorescence in 293T cells, similar to the positive control anti-Meq mAb FD7, whereas the supernatant of hybridoma 4F3 did not show any red fluorescence. The non-specific reaction of candidate Meq antibodies or positive control FD7 was simultaneously excluded by the IFA staining of 293T cells that were transfected with the empty plasmid pEGFP-N1 (Figure 3). Taken together, the data suggests that except for 4F3, hybridomas 2A9, 5A7, 7F9 and 8G11 secreted specific antibodies against the Meq proteins.

### 3.4. Confocal Analysis of the Expression of Meq Using Meq-Specific Antibodies

**Figure 4:**
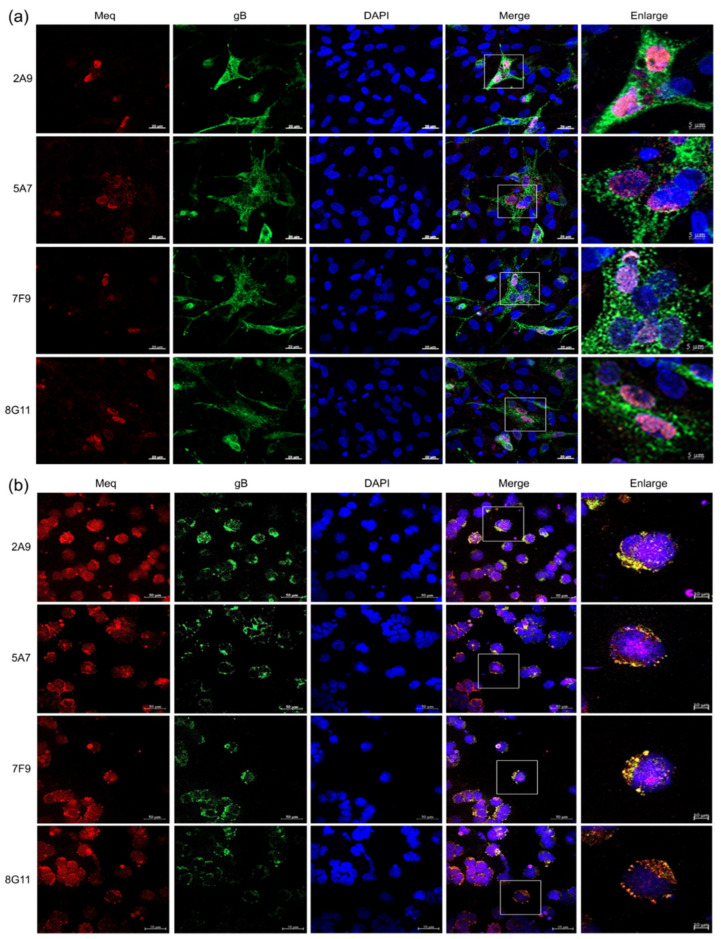
Confocal analysis of the Meq and gB proteins in MDV-infected CEFs and transformed MSB-1 cells. (**a**) Staining of the Meq and gB proteins in virus-infected CEF cells. The GX0101-infected CEFs were trypsinized, diluted and transferred to a special confocal culture dish. Then, the cells were fixed and incubated with Meq-specific antibodies and DyLight 594 labeled Goat Anti-Mouse IgG sequentially, followed by the incubation with anti-gB mAb HB3 and DyLight 488 labeled Goat Anti-Mouse IgG, respectively. The DAPI was used to stain and indicate the nuclei of CEF cells. Meq, MDV−1 oncoprotein stained in red; gB, MDV−1 glycoprotein B stained in green; DAPI, 4’,6-diamidino-2-phenylindole used to indicate the nuclei of cells in blue; Merge, merged image in purple; Enlarge, enlarged pictures from white brackets in merged images. (Scale bar = 20 µm or 5 µm) (**b**) Staining of the Meq and gB proteins in MSB-1 cells. The cells were adhered to the confocal special cell culture dish and the IFA staining was performed in the same way. Captions are the same as described above. (Scale bar = 50 µm or 10 µm).

Utilizing confocal analysis, we have further analyzed the intracellular localization of Meq stained by newly generated Meq-specific antibodies in GX0101-infected CEFs and MDV-transformed MSB-1 cells. As expected, the Meq protein was stained in red by the supernatant of all of the four hybridomas 2A9, 5A7, 7F9 and 8G11 with a nuclear distribution, whereas gB proteins stained with the anti-gB mAb HB3 in green localized in cytoplasm (Figure 4A). The confocal analysis results of MSB-1 cells were completely similar to that of the MDV-infected CEF cells, all showing the specific Meq stained in red in the nucleus (Figure 4B).

### 3.5. Purification and Subtype Identification of Meq-Specific mAbs

For further development of purified mAbs against the Meq proteins, the hybridomas 2A9 and 8G11 were single cell cloned by limiting dilution and two purified clones, namely 2A9-B12 and 8G11-B2, were selected to prepare ascitic fluids from Balb/C mouse. The subtypes of mAbs 2A9-B12 and 8G11-B2 were characterized to be IgG2b/Kappa or IgG1/Kappa as determined using the Mouse Monoclonal Antibody Isotyping Kit. The reactivity of ascitic fluids of mAbs 2A9-B12 and 8G11-B2 remained positive even up to the dilutions of 1:10,000 and 1:4000, respectively, as determined by IFA staining. 

### 3.6. Reaction Spectrum of the Meq-Specific mAbs 2A9-B12 and 8G11-B2

**Figure 5:**
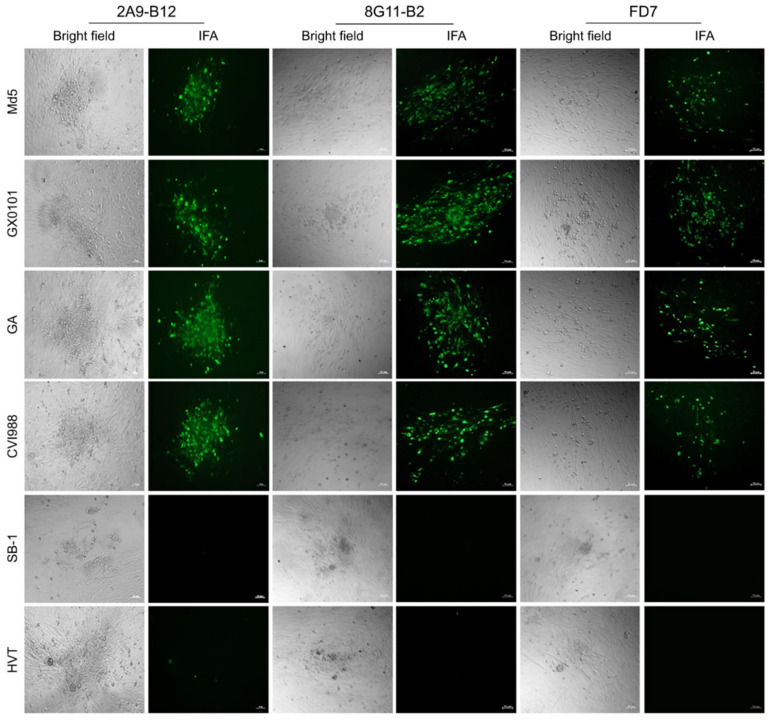
Cross reactions of mAbs 2A9-B12 and 8G11-B2 to the viral plaques produced by different types of MDVs detected by IFA staining. The reaction spectrums of newly developed Meq mAbs to different types of MDV and HVT were determined by IFA staining. The anti-Meq mAb FD7 served as a positive control. Bright field, images of virus plaques under regular light; IFA, immunofluorescence assay. (Scale bar = 50 µm).

**Figure 6:**
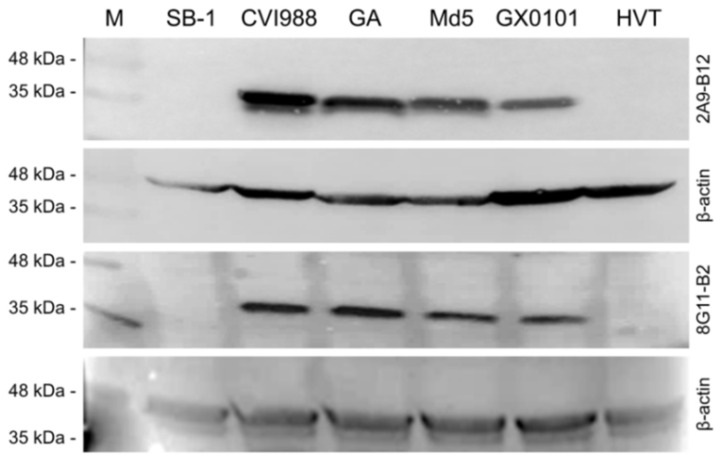
Western blot analysis of reactivity of mAbs 2A9-B12 and 8G11-B2 to the Meq proteins from MDV strains with different virulence. The reaction spectrums of newly developed Meq mAbs to different types of MDV and HVT were determined by Western blot analysis. Meq, MDV−1 oncoprotein. The chicken β-actin was used as protein loading control. M, protein molecular weight marker.

For further characterization of the newly developed Meq-specific mAbs, reaction spectrums of mAbs 2A9-B12 and 8G11-B2 against different types of MD associated avian herpesviruses were determined by both IFA and Western blot analysis. As demonstrated in Figure 5, the viral plaques in CEF monolayers produced by infection of different MDV−1 strains, including Md5, GX0101, GA and CVI988, have been specifically stained with green fluorescence after incubation with mAbs 2A9-B12 or 8G11-B2, while the viral plaques produced by HVT or SB-1 viruses did not show any fluorescence staining. Western blot analysis also showed the similar results to that of IFA staining. As demonstrated in Figure 6, none of specific reaction bands was observed in the samples collected from HVT or SB-1-infected CEF cells but in those collected from CEF cells infected with MDV−1 strains, a strong signal of protein bands with a molecular weight about 40 kDa was observed as expected, consistent with the molecular size of the MDV−1 specific Meq protein.

## 4. Discussion

Generation of MDV-specific antibodies is crucial for studying MDV pathogenesis and oncogenesis. However, it is generally difficult to obtain ideal mAbs against viral proteins, and this has been particularly true for the oncoprotein Meq. In the present study, we have generated Meq-specific mAbs using KLH-coupled synthetic polypeptides derived from the conserved and hydrophilic regions of Meq as immunogens. In the initial stage of mAb screening by IFA staining, we have identified a total of five positive hybridomas 2A9, 4F3, 5A7, 7F9 and 8G11 secreting specific antibodies that reacted with MDV plaques. For further improving the specificity of the positive hybridomas, we have performed IFA cross screening with two Meq-deleted MDV mutants Md5∆meq and GX0101∆meq previously generated utilizing CRISPR/Cas9-based gene editing system [24], together with the parental viruses Md5 and GX0101. Using this approach, we could easily identify the four hybridomas 2A9, 5A7, 7F9 and 8G11 that were indeed targeting Meq. The specificity of each of the hybridomas was further confirmed by positive IFA staining of Meq-overexpressing 239T cells by these antibodies. In addition, we also observed a false-positive hybridoma 4F3 from initial screening. It may be due to that the Meq polypeptide used as immunogen is relatively short, only 30 to 45 amino acids, which may have a homology with some certain amino acid segment of elsewhere in other MDV proteins. In conclusion, our data indicates that using the synthesized polypeptide immunization combined with cross IFA screening of CRISPR/Cas9 gene-edited viruses is a new efficient strategy for the generation and identification of specific antibody against viral proteins, such as Meq. 

For the immunogens presently used, four synthetic polypeptides were designed mapping to the nuclear localization region, Leucine Zipper and transactivation domain of Meq, respectively. Interestingly, we found that only some of the positive hybridomas, such as 7F9 and 8G11, secrete Meq-specific antibodies and recognize the Meq polypeptide 1. However, positive staining by all the mAbs in confocal microscopic analysis has demonstrated the nuclear localization of Meq in both of the virus-infected CEFs and MDV-transformed MSB-1 cells, which is in accordance to the position of the polypeptide 1 within the nuclear localization region of BR1 in the N-terminal of Meq. In a previous work [25], a Meq-specific mAb named as 23B46 was also found to recognize the BR1 domain in the nuclear localization region. Considering the critical role of Meq in triggering MD tumorigenesis, it deserves to further identify the accurate antigenic epitope recognized by these antibodies for future studies.

During the golden age of mAb development from the 1980s to 1990s, using homogenization or sonication of the repeatedly frozen and thawed virus-infected CEF cultures as immunogens, several MDV specific hybridomas have been developed, but identities of most of hybridomas remained unclear due to the limitations in experimental technology at that time [16,17,18,19]. In the late 1990s, with the development of new molecular biological techniques, a number of specific mAbs against MDV proteins such as gB, pp38, gD, gp82, ICP4 and ORF873 have been successively developed using recombinant proteins expressed in baculovirus system as immunogens [26,27,28,29,30,31]. Due to the low level expression of Meq in MDV-infected CEF cells, recombinant proteins were used as immunogens for developing all existing mAbs of Meq [20,21,22,23]. It is generally known that the Meq gene have a high mutation rate, such as insertion or deletion in the C-terminal proline-rich region, or point mutations in some fixed sites [32,33,34]. The cross reactivity of anti-Meq mAbs previously prepared with recombinant proteins as immunogens had not been fully investigated, and most of them has only been applied in IFA staining or IHC analysis [20,21,22,23]. Herein, we have successfully produced Meq-specific mAbs using synthetic polypeptide from the conserved and hydrophilic region of Meq. The newly developed mAbs 2A9-B12 and 8G11-B2 have been demonstrated to have a wide spectrum to react with different virulent MDV−1 strains, such as Md5, GX0101, GA and vaccine strain CVI988, and are applicable not only for IFA staining but also for Western blot and confocal analysis. In future work, more applications of these new Meq-specific mAbs in the diagnosis technology and fundamental research of MD biology deserve to be explored. 

The CRISPR/Cas9-based system is a new generation of gene editing technology and has been widely applied in virology research on the large DNA viruses, especially the family of herpesviruses [35]. As for avian herpesviruses including MDV, great advances have been achieved in the functional studies of both protein-coding genes and non-coding RNAs, and even for the development of innovative MD vaccines [36,37,38,39,40,41,42,43]. In a latest work [44], we have first demonstrated a new strategy for the efficient cross-screening and identification of mAbs against MDV−1-specific pp38 proteins, from a pool of mAbs against MDV, by IFA staining with the parental and mutant viruses generated utilizing the CRISPR/Cas9-based gene-editing technology. It has provided a meaningful reference for the future generation of antibodies against viruses, especially for large DNA viruses such as herpesviruses. However, the Meq-specific mAbs had not been obtained from this work using the same way. It is possibly due to the low-level expression and nuclear localization of Meq in MDV-infected CEFs, which could not induce enough positive hybridomas for further screening and identification. Thus, in the present work, we have resolved two key problems, the low-level expression of immunogen and rapid screening of positive hybridoma, for the efficient development of Meq-specific mAbs. In conclusion, we have displayed a new successful strategy to generate Meq-specific mAbs using synthesized hydrophilic polypeptide as immunogen combined with the CRISPR/Cas9-based screening technology, which has provided a new efficient approach for future generation of virus protein-specific mAbs.

## Figures and Tables

**Table 1:** Sequences of Meq polypeptides synthesized for making antigens.

Polypeptide	Sequence	Length (aa)	Origination
**1**	aa_26_GSTSRRKKRKSHDIPNSPSKHPFPDGLSEEEKQKLERRRKRNRDAaa_70_	45	nuclear localization region
**2**	aa_81_YVDKLHEACEELQRANEHLRKEIRDLRTECTSLaa_113_	33	Leucine Zipper
**3**	aa_143_PVPEPPICTPPPPSPDEPNAPHCSGSQPPICTPaa_175_	33	transactivation domain
**4**	aa_285_LFTPSPPHPAPEPERLYARLTEDPEQDSLYSaa_315_	31	transactivation domain

## Data Availability

Not applicable.
